# Narcissism and Entrepreneurial Well-Being: The Moderating Role of Equity Ranking and Industry Attention

**DOI:** 10.3390/bs15111438

**Published:** 2025-10-22

**Authors:** Yiran Liu, Xiaoya Liang, Chengying Zhang, Wei Dong, Xin Li, Huihui Li

**Affiliations:** 1 School of Education, Tianjin University, Tianjin 300350, China; yiran.liu@tju.edu.cn (Y.L.); liangxiaoya@tju.edu.cn (X.L.); zhangchengying@tju.edu.cn (C.Z.); 2 College of Management and Economics, Tianjin University, Tianjin 300072, China; lixin131516@tju.edu.cn; 3 School of Business, Nanjing Normal University, Nanjing 210023, China

**Keywords:** entrepreneurship, well-being, narcissism, equity ranking, industry attention

## Abstract

Why do some entrepreneurs experience higher levels of well-being? While prior research has extensively investigated various determinants of entrepreneurial well-being, this study advances the literature by examining the unique role of narcissism in shaping entrepreneurs’ well-being. We propose that a narcissistic personality—characterized by heightened self-focus and need for recognition—interacts with contextual factors to enhance well-being. Specifically, narcissistic entrepreneurs’ well-being increases when equity ranking is high and industry attention is salient. Using a multi-method approach, combining survey data from 165 Chinese entrepreneurs with archival data, we demonstrate that narcissism positively predicts well-being, with effects amplified by both high equity ranking and industry attention. In doing so, this study contributes to research on well-being and narcissistic personality in entrepreneurial contexts.

## 1. Introduction

Entrepreneurship plays a crucial role in social and economic development (Rindova et al., 2009). While its financial value is widely acknowledged, entrepreneurs’ mental well-being significantly affects their capacity to innovate and stimulate economic growth (Stephan, 2018; Kraus et al., 2020). Therefore, understanding the factors that influence entrepreneurs’ well-being is essential.

Well-being refers to a state of mental health marked by positive emotions, vitality, meaning, and self-realization (Deci et al., 2001; Keyes, 2006). In entrepreneurship, research has debated whether entrepreneurs experience greater well-being (Burger et al., 2024). Some argue that the autonomy and control in entrepreneurship enhance well-being (Hyytinen et al., 2013; Hamilton, 2000), while others point to the stressors and high risks that may undermine it (Stephan, 2018). This divergence raises key questions: Why do some entrepreneurs report higher well-being, and under what conditions do they feel fulfilled?

Studies have analyzed entrepreneurial well-being at environmental, organizational, and individual levels (Abreu et al., 2019; Xiong et al., 2020; Xu et al., 2021). Given its subjective nature, the individual level has drawn particular interest (Kahneman & Krueger, 2006). Prior work often links well-being to the fulfillment of goals or intrinsic needs (Cooper & Artz, 1995; Ryan & Deci, 2001; Uy et al., 2013; Stephan et al., 2020). Yet entrepreneurs vary in motivations—some seek self-realization, others financial gain (Block & Wagner, 2010; Astebro, 2017). Thus, well-being likely depends on how well the entrepreneurial context aligns with their personal needs.

Entrepreneurs’ needs are influenced by their distinctive traits. In particular, narcissism is a crucial trait among entrepreneurs (D. Miller, 2015). Narcissists have an inflated self-view and tend to believe that they have superior abilities over others (Judge et al., 2006). They often seek constant attention and admiration from others, which further reinforces their elevated self-view (Chatterjee & Hambrick, 2007). Entrepreneurship satisfies the narcissist’s inherent need for attention and external validation (Navis & Ozbek, 2016). Additionally, narcissists possess a strong desire for dominance and leadership (Farwell & Wohlwend-Lloyd, 1998; Emmons, 1987). Entrepreneurship grants them decision-making autonomy and control, enabling them to exert influence and fulfill their need for superiority, thereby enhancing their sense of achievement and psychological satisfaction. Consequently, entrepreneurship simultaneously satisfies narcissistic entrepreneurs’ core needs for “attention” and “control,” which significantly enhances their well-being.

The extent to which entrepreneurs’ needs are satisfied also depends on contextual factors. This study primarily examines the moderating effects of entrepreneurial equity ranking and industry attention on this relationship. Equity represents the privileges granted to shareholders by virtue of their ownership in a company (Lerner & Leamon, 2023). A higher equity ranking implies greater control and autonomy in decision-making, thereby more fully satisfying narcissistic entrepreneurs’ inherent need for power and dominance (Wasserman, 2017). At the same time, highly narcissistic entrepreneurs possess a profound desire for attention (Emmons, 1984) and often exhibit behaviors aimed at attracting it (Kernberg, 1974). Greater industry attention provides narcissistic individuals with more opportunities to gain external recognition and showcase their abilities, thereby reinforcing their sense of self-worth.

Notably, while leadership roles matter, this study centers on the individual psychological level—specifically, how narcissism as a personality trait influences entrepreneurs’ subjective well-being, rather than leadership effectiveness or venture outcomes. Entrepreneurship offers a unique setting for narcissists to fulfill core needs like attention and control. Moreover, focusing on Chinese entrepreneurs adds theoretical relevance. In China’s culture, which emphasizes social evaluation and collective achievement (Li et al., 2015), narcissistic individuals may be especially sensitive to status (e.g., equity ranking) and reputation (e.g., industry attention).

We combined survey data from 165 entrepreneurs with archival data on equity rankings and industry attention to examine how narcissism affects entrepreneurial well-being. This multi-method approach reveals how objective contextual factors moderate this relationship, advancing beyond prior self-report studies.

## 2. Literature Review

### 2.1. Well-Being

Based on the character strengths theory proposed by Peterson and Seligman (2004), this study adopts an integrated perspective to define well-being. Current research on well-being is primarily grounded in two theoretical frameworks: hedonic and eudaimonic well-being. Hedonic well-being emphasizes the pursuit of pleasure and the avoidance of pain, positing that well-being is characterized by the presence of positive affect and the absence of negative affect (Kahneman et al., 1999). In contrast, eudaimonic well-being transcends mere pleasure, conceptualizing well-being as a state in which individuals realize their true potential (Ryan et al., 2008). This perspective encompasses elements such as a sense of accomplishment, self-expressiveness (Waterman et al., 2010), self-actualization (Maslow, 1968), and personal growth (Erikson, 1994). Accordingly, this study defines well-being as a state of mental health that incorporates positive emotional experiences, physical and mental vitality, a sense of meaning in life, and self-actualization (Kahneman et al., 1999; Ryan et al., 2008; Maslow, 1968).

Cultivating well-being is vital for sustainable entrepreneurship. Consequently, the concept of entrepreneurial well-being has garnered many research interests. Macro-level research has examined how entrepreneurial ecosystems, sociocultural factors, and other contextual elements influence the well-being of entrepreneurs (Volkmann et al., 2021; Méndez-Picazo et al., 2021; Deci et al., 2001; Shir, 2015). However, well-being is inherently subjective among entrepreneurs (Kahneman & Krueger, 2006). By investigating individual differences, we can identify pivotal factors that shape entrepreneurial well-being, allowing for more personalized support and effective intervention strategies. As a result, understanding individual heterogeneity in entrepreneurial well-being has emerged as a central research focus. Current research adopts a cognitive perspective to assess the relationship among entrepreneurs’ stress perceptions, their coping mechanisms, and overall well-being. For instance, optimistic outlooks on the future (Lindblom et al., 2020) and the adoption of positive stress-coping strategies (Uy et al., 2013) contribute to enhanced well-being. However, the eudaimonic perspective emphasizes that well-being transcends mere cognitive regulation for conflict reduction and emotion boosting. It is about realizing one’s full potential, fulfilling personal values, and satisfying innate needs, as Ryan et al. (2008) articulate.

The intrinsic drive for autonomy and self-actualization motivates many individuals to pursue entrepreneurial ventures. This intrinsic desire has led researchers to investigate whether entrepreneurship enhances well-being compared to other professions. Findings indicate that entrepreneurship can improve well-being by satisfying individuals’ needs for autonomy (Ryan & Deci, 2001), achievement (Uy et al., 2013), and purpose (Stephan et al., 2020). Furthermore, research shows that the well-being of entrepreneurs fluctuates throughout the entrepreneurial process, influenced by demand magnitude and motivation orientation. Entrepreneurs who encounter lower demand often report higher satisfaction and overall well-being, enjoying greater well-being when they set modest financial expectations or consistent goals (Cooper & Artz, 1995). Additionally, studies indicate that internally motivated entrepreneurs tend to experience higher levels of well-being than their externally motivated counterparts (Lyubomirsky, 2001).

Research has shown that entrepreneurs are more likely to experience higher well-being when their entrepreneurial needs and motivations are satisfied. However, these needs and motivations exhibit heterogeneity. This raises two issues: First, is well-being different for entrepreneurs with different personality traits? Second, how can the entrepreneurial context adequately fulfill these persistent needs? Entrepreneurs possess various motives that may stem from their personality traits. The relationship between these traits and their well-being is a topic that warrants further exploration. Even when these needs and motives are specific and stable, the entrepreneurial context may not adequately fulfill them. More research is needed to understand how personal traits and situational factors interact to affect well-being. Accordingly, this study aims to examine the relationship between the narcissistic personality of entrepreneurs and well-being, as well as how situational characteristics moderate this relationship.

### 2.2. Narcissism and Well-Being

Narcissism is a crucial personality trait among entrepreneurs, characterized by an exaggerated sense of self-worth and a dependence on external validation for self-affirmation (Raskin & Terry, 1988; Sedikides et al., 2019). The study focuses on “subclinical narcissism” or “trait narcissism”—a personality dimension that varies across individuals in the general population (Cain et al., 2008; J. D. Miller & Campbell, 2008). This differs fundamentally from Narcissistic Personality Disorder (NPD), which is a clinically diagnosed mental disorder meeting specific diagnostic criteria and causing significant functional impairment (Pincus & Lukowitsky, 2010). This trait encompasses two fundamental dimensions. In terms of cognition, narcissists display intense self-absorption and an exaggerated perception of their capabilities (Judge et al., 2006). In terms of motivation, they continuously seek attention and affirmation from others to maintain their self-esteem (Chatterjee & Hambrick, 2007). As a result, individuals with pronounced narcissistic traits often pursue positive validation and enjoy challenging tasks (Wallace & Baumeister, 2002). Thus, entrepreneurship serves as an ideal context for studying narcissistic behavior. Previous studies have predominantly explored the relationship between narcissistic personality traits and entrepreneurial behaviors (Leung et al., 2021; Burger et al., 2024). It revealed that narcissism is positively associated with entrepreneurial intention (Hmieleski & Lerner, 2016), entrepreneurial entry (Mathieu & St-Jean, 2013), and entrepreneurial orientation (Wales et al., 2013a) and well-being (Leung et al., 2021). Collectively, these findings suggested that narcissistic personality traits serve as a significant motivator for entrepreneurial activity (Navis & Ozbek, 2016). Notably, this study posits that narcissism not only fosters the initiation of entrepreneurial efforts but also enhances the well-being of entrepreneurs.

This enhancement occurs because entrepreneurship fulfills two key needs for highly narcissistic entrepreneurs more than for those with lower narcissism. First, highly narcissistic entrepreneurs have a strong need for external attention and recognition, which reinforces their self-esteem (Morf & Rhodewalt, 2001). While they exhibit confidence in their abilities (Campbell et al., 2002), their self-esteem remains sensitive and reliant on external validation (Morf & Rhodewalt, 2001). In the entrepreneurial context, they find ample opportunities to showcase their talents and achieve the acknowledgment that bolsters their self-worth (Navis & Ozbek, 2016). The pursuit of recognition elevates self-esteem and fosters positive emotional experiences, providing individuals with a meaningful sense of purpose in chasing their passions (Orth & Robins, 2022). Additionally, the entrepreneurial challenges they face contribute to both physical and mental vitality, as engaging in demanding tasks facilitates their journey toward self-fulfillment (Zeigler-Hill, 2006). Collectively, these aspects of entrepreneurship play a significant role in enhancing their well-being (Rhodewalt & Morf, 1998).

Second, highly narcissistic entrepreneurs exhibit a strong desire to lead in interpersonal dynamics (Farwell & Wohlwend-Lloyd, 1998). They believe they are entitled to lead decision-making processes (Farwell & Wohlwend-Lloyd, 1998). This inclination often leads them to dominate and manipulate those around them (Emmons, 1987). In the context of startups, entrepreneurs typically possess significant decision-making authority and autonomy, which further nurtures their desire for control. Consequently, this environment allows narcissistic individuals to exert their influence and fulfill their craving for superiority. As they navigate the challenges of entrepreneurship and assert their leadership, they not only enhance their sense of achievement but also promote their well-being. The ability to lead effectively provides them with a heightened sense of purpose and fulfillment, ultimately contributing to their overall psychological satisfaction.

**Hypothesis** **1.** *The level of narcissism in entrepreneurs is positively correlated with well-being.*

### 2.3. The Moderating Role of Entrepreneurial Equity Ranking

The previous discussion suggests that narcissistic entrepreneurs may experience heightened well-being due to the external attention and autonomy that entrepreneurship provides. However, the degree of fulfillment of these needs is contingent on contextual factors. This study examines the moderating effects of entrepreneurial equity rankings and industry attention on this relationship.

Entrepreneurship enhances individual control over the business. However, the extent of such control is influenced by the entrepreneur’s position within the corporate hierarchy. Equity represents the privileges afforded to shareholders by virtue of their ownership in a firm (Lerner & Leamon, 2023) and serves as a crucial indicator of entrepreneurial authority. Equity is divided into self-beneficial rights and co-beneficial rights. The former includes dividends and bonuses based on profitability, and the latter includes voting and supervisory rights (Lerner & Leamon, 2023). Generally, a larger equity share corresponds to greater decision-making authority and control.

This study analyzes equity rankings to encapsulate the entrepreneur’s position and influence within the firm (Wasserman, 2017). Entrepreneurs with higher equity possess substantial authority over critical decisions, thereby enhancing their control. Specifically, those at the top of the equity ranking hierarchy tend to exert significant influence over strategic direction, satisfying the needs of narcissistic entrepreneurs and improving their well-being. Conversely, those ranking lower in equity may face limitations in decision-making, hindering their ability to dominate and diminishing their well-being. Therefore, this study proposes that entrepreneurial equity ranking moderates the relationship between narcissism and well-being.

**Hypothesis** **2.** *Entrepreneurial equity ranking moderates the relationship between narcissism and well-being. The positive relationship between narcissism and well-being intensifies when the entrepreneur’s equity ranking is high.*

### 2.4. The Moderating Role of Industry Attention

The public attention received by entrepreneurs often extends beyond their firms to encompass broader industry and societal contexts. This study introduces the concept of “industry attention”, defined as the degree of public interest and engagement directed toward a specific industry (Croteau & Hoynes, 2006). When industry attention increases, it often results in heightened social attention, as evidenced by greater media coverage and rising search volumes on social media platforms. As societal demands and policy priorities shift, industries may experience varying degrees of external attention over time. For example, during the COVID-19 pandemic, the biopharmaceutical sector captured substantial public interest, leading to intensified media coverage and discussions.

Highly narcissistic entrepreneurs possess a profound desire for attention (Emmons, 1984) and frequently exhibit behavior to attract it (Kernberg, 1974). Entrepreneurs operating within highly visible industries are more likely to receive media exposure and public interest. This environment provides narcissistic entrepreneurs with a chance to show their competencies, fulfilling their need for personal recognition and enhancing well-being. Additionally, narcissists experience boosts to their self-esteem when their actions are publicly noticed (Wallace & Baumeister, 2002). As a result, the well-being of narcissistic entrepreneurs may be positively impacted by increases in industry attention. This attention may not only motivate them to excel in their industry but also inspire them to strive for leadership. In this context, the entrepreneurial serves to amplify their self-esteem and emotional satisfaction, leading to a heightened sense of purpose. Accordingly, this study posits that industry attention moderates the relationship between narcissism and well-being. When industry attention is robust, highly narcissistic entrepreneurs are likely to feel more validated and recognized, further reinforcing their self-worth. This dynamic promotes an even greater sense of achievement and fulfillment. Ultimately, it increases their well-being.

**Hypothesis** **3.** *Industry attention moderates the relationship between narcissism and well-being. The strength of the positive association between narcissism and well-being is amplified under conditions of heightened industry attention.*

## 3. Method and Materials

### 3.1. Data and Sample

This study investigates the well-being of entrepreneurs in China. It is a rapidly growing market characterized by its unique economic dynamics and cultural context. The Chinese market, with its enormous domestic demand and burgeoning economic potential, offers entrepreneurs ample opportunities for growth and expansion. Simultaneously, the Chinese government is promoting innovation and entrepreneurship, nurturing a supportive environment outside of startups. Additionally, the entrepreneurial landscape in China showcases a vibrant mix of diverse participants. Understanding the experiences of entrepreneurs in such a vibrant environment is crucial for both theoretical and practical insights, particularly as China increasingly influences global economic trends.

The data were collected from entrepreneurs across various regions in China. To ensure data quality, this study distributed and collected paper-based questionnaires at national-level business incubators located in Eastern and Southeastern China. To minimize sample selection bias, the research team adopted a random sampling method. Within these incubators, enterprises were approached systematically based on spatial order (from near to far, from low to high floor), selecting one enterprise for every five encountered. If a business manager was unavailable or declined to participate, the team proceeded to the next enterprise and continued the pattern—skipping five more enterprises before attempting the next survey. To encourage participation and improve the response rate, a monetary incentive was offered to respondents who completed the questionnaire.

Clear inclusion and exclusion criteria were established to ensure sample homogeneity. Inclusion criteria required participants to be founders of the enterprise (holding equity and involved in major business decisions). Exclusion criteria included enterprises established for less than six months and participants not involved in core decision-making. A total of 300 questionnaires were distributed, and 221 were returned. After data cleaning—which involved removing responses that were incomplete, inattentive, or lacked key information—and cross-verifying equity data using Qichacha archival records to ensure accuracy, 165 valid questionnaires were retained, resulting in an effective response rate of 74.7%.

Our sample of entrepreneurs is geographically diverse, encompassing cities in eastern and southern China, including Beijing, Xiamen, Guangzhou, Qingdao, Jinan, and Zibo. Of the participants, 78.2% are male, and 48.5% are aged between 26 and 40. Additionally, 53.3% of respondents hold bachelor’s degrees, and 57.6% are first-time entrepreneurs. Detailed demographic information and basic firm-level descriptors are summarized in Table 1.

### 3.2. Common Methodology Bias Analysis

Common method bias (CMB) refers to the spurious correlation between predictor variables and validity variables. These biases can arise from shared data sources, raters, measurement contexts, item framing, and the inherent properties of the items (Podsakoff et al., 2003). To mitigate potential CMB effects, we employed a multifaceted methodological approach. First, the study utilized an anonymous survey methodology to reduce subjective influences and minimize social desirability bias, enhancing the accuracy of responses. Second, we adopted a pluralistic data collection strategy to prevent over-reliance on a single data source. To mitigate common method bias, we employed a multi-source design by collecting subjective data through self-reported surveys (measuring narcissism using the NPI-16 and well-being via Likert scales) and objective archival data (equity rankings from Qichacha and industry attention from the Baidu Index). This separation of data sources helps reduce spurious correlations and enhances the validity of our findings. Notably, both narcissism and well-being were based on subjective self-reports. The narcissism scale was structured as a forced-choice questionnaire, whereas the well-being scale was administered using a Likert 7-point scale. Finally, we conducted the Harman one-factor test to assess potential common method bias. This analysis extracted eight factors from the unrotated components, with the first factor accounting for only 23.7% of the variance. This indicates that no single factor dominated the variance, suggesting that significant homoscedasticity issues were absent.

### 3.3. Variable Measurements

#### 3.3.1. Dependent Variable: Well-Being

The measurement of well-being utilized in this study was adapted from Spector’s (1985) original construct. This included additional items deemed highly relevant to the entrepreneurial domain, such as self-actualization and positive emotional states. We employed a 7-point Likert scale, with responses ranging from “strongly disagree” (1) to “strongly agree” (7). The scale consisted of ten items, such as “I enjoy entrepreneurship”, “I take pride in my entrepreneurial endeavors”, “During entrepreneurship, I have felt satisfaction”, and “I am enthusiastic about entrepreneurship”. The Cronbach’s α coefficient for this scale was 0.908, indicating a high level of internal consistency and reliability.

#### 3.3.2. Independent Variable: Narcissism

This study employed the short-form Narcissistic Personality Inventory (NPI-16) to measure core narcissistic traits, which reflect a general tendency characterized by the pursuit of attention, admiration, and affirmation. We draw on Ames et al. (2006) to highlight the advantage of the NPI-16 as an efficient tool for capturing overall narcissism levels, which is highly relevant to our investigation of the relationship between narcissism and well-being. The NPI-16 was specifically developed and validated to measure non-clinical narcissistic traits in general populations (Ames et al., 2006).

The NPI-16 was widely recognized as a valid and reliable instrument for measuring narcissistic dimensions (Gentile et al., 2013; Ames et al., 2006) and used in entrepreneurship research (Wales et al., 2013b). The NPI-16 employs a forced-choice format consisting of 16 paired items, where participants choose the statement that best reflects their self-perception. For example, there are two perspectives: (a) I view myself as an extraordinary person, and (b) I see myself as pretty much like everyone else. Response (a) is coded as 1, while response (b) is coded as 0. The selection of response (a) is scored as 1, while response (b) is scored as 0. The average score across the items is calculated, with higher scores indicating greater narcissistic traits. The Cronbach’s α coefficient for the scale was 0.721, indicating a respectable level of reliability.

#### 3.3.3. Moderating Variable: Equity Ranking

Entrepreneurs’ equity ranking was measured using archival data from Qichacha, which provides verified records of shareholders and their equity stakes. We categorized equity ranking as a binary variable (top equity ranking = 1, non-top equity ranking = 0) based on the entrepreneur’s ownership percentage relative to other shareholders. Self-reported equity rankings were cross-validated with Qichacha records to ensure consistency.

#### 3.3.4. Moderating Variable: Industry Attention

This study focuses on science and technology entrepreneurship across nine industries: the new generation of information technology industry, software and information technology services industry, new materials industry, high-end equipment manufacturing industry, biotechnology industry, new energy industry, business services industry, science and technology promotion and application services industry, among others.

Industry attention was measured using archival data from the Baidu Index, which quantifies public interest through search frequencies. We collected daily search volumes for nine industries (e.g., new energy, biotechnology) from January 2018 to April 2021. Industry attention was operationalized as a binary variable (high = 1, low = 0) based on aggregate search volumes. To measure industry attention accurately, we identified specific keywords for each industry. We used third-tier names from the “Industrial Classification for national economic activities” and the second-tier names from the “Classification of Strategic Emerging Industries”. We then calculated the aggregate search volumes for each industry using a formula to quantify industry attention. Details are provided in Table 2. In the formula, “St” represents total industry attention for industry “t”, “N” is the number of industry-specific keywords, and “Si,t” is the search volume of the “i”-th keyword for industry “t”.

$$S_{t}=\frac{1}{N}\sum_{i=1}^{N}S_{i,t}$$

Among the nine industries, the new energy industry, the new generation of information technology industry, and the new materials industry exhibited the highest attention levels, each scoring above 500. To highlight the differences in attention, we categorized these as high-attention industries, coded as 1, while the remainder were classified as low-attention industries, coded as 0.

#### 3.3.5. Control Variables

To rigorously examine the relationship between narcissism and well-being in the entrepreneurial context, it is essential to consider the influence of individual traits, entrepreneurial experience, and the organizational structure of the entrepreneurial team. Therefore, this study identifies control variables across three levels.

Individual Level: Personal characteristics are pivotal determinants of well-being. Educational attainment correlates positively with well-being (Block & Wagner, 2010). Furthermore, higher self-efficacy is linked to improved well-being (Di Fabio & Palazzeschi, 2009), and empathy is associated with greater life satisfaction (Grühn et al., 2008). To account for these variables, we included controls for education (high school/less than secondary school = 1, college = 2, bachelor’s degree = 3, master’s degree = 4, doctorate = 5), self-efficacy, and empathy. Measuring self-efficacy and empathy through Likert questions. Furthermore, prior research has shown that well-being may be influenced by entrepreneurial experience (Uy et al., 2013) and working experience (Stephan, 2018). Therefore, we included the number of entrepreneurial ventures and work experience as control variables. Entrepreneurial experience was measured by the number of new ventures the entrepreneur has created. Working experience was measured by whether or not the entrepreneur had worked for a large organization.

Organizational Level: Performance metrics such as profit growth rates directly impact well-being, reflecting increased job satisfaction in high-performing entrepreneurial teams (Diener & Seligman, 2004). Additionally, well-being varies across different types of entrepreneurships (Svetek & Drnovsek, 2022). Hence, we incorporated performance and types of entrepreneurships as control variables in this study.

#### 3.3.6. Confirmatory Factor Analysis

To examine the discriminant validity of the core constructs, we conducted confirmatory factor analyses (CFA) for the narcissism and well-being scales, respectively. The results showed a good model fit for the narcissism scale: χ^2^/df = 1.186 < 5, SRMR = 0.061 < 0.08, RMSEA = 0.034 < 0.08, IFI = 0.946 > 0.9, TLI = 0.926 > 0.9, CFI = 0.941 > 0.9. The well-being scale also demonstrated a good model fit: χ^2^/df = 1.531 < 5, SRMR = 0.035 < 0.08, RMSEA = 0.057 < 0.08, IFI = 0.983 > 0.9, TLI = 0.974 > 0.9, CFI = 0.983 > 0.9. All indices met or exceeded the recommended academic thresholds, indicating that both scales exhibited good construct validity in this study.

## 4. Results

### 4.1. Descriptive Statistics and Correlation Analysis

Descriptive statistics and correlations for all variables are presented in Table 3. The analysis revealed a significant positive correlation between narcissism and well-being (r = 0.349, *p* < 0.01). This result is consistent with Hypothesis 1, indicating a positive association between narcissistic traits and entrepreneurial well-being in our sample. The correlations among other variables were generally low to moderate, reducing concerns about multicollinearity in subsequent analyses.

### 4.2. Research Hypothesis Validation

The proposed hypotheses were tested using hierarchical regression analysis in IBM SPSS Statistics, Version 24.0, (Manufacturer: International Business Machines (IBM) Corporation; Manufacturer Location: Armonk, NY, USA) and the results are summarized in Table 4. The analysis comprised three models: Model 1 included only control variables. Subsequently, Model 2 expanded on the previous model by incorporating narcissism, equity ranking, and industry attention as new independent variables. Lastly, Model 3 included interaction terms to explore how narcissism interacts with equity ranking and industry attention.

Supporting Hypothesis 1, a significant positive association was observed between narcissism and well-being (β = 0.370, *p* < 0.01). This indicates that higher levels of narcissism are linked to higher levels of well-being in this cross-sectional sample.

Model 3 examined the moderating effects by adding the interaction terms. The results supported Hypothesis 2, as the interaction between narcissism and equity ranking was marginally significant (β = 0.123, *p* < 0.1). This signifies that the positive association between narcissism and well-being is stronger for entrepreneurs with a top equity ranking. Similarly, the marginally significant interaction for narcissism and industry attention (β = 0.128, *p* < 0.1) supported Hypothesis 3, indicating that the association is more pronounced in high-attention industries.

**Figure 1:**
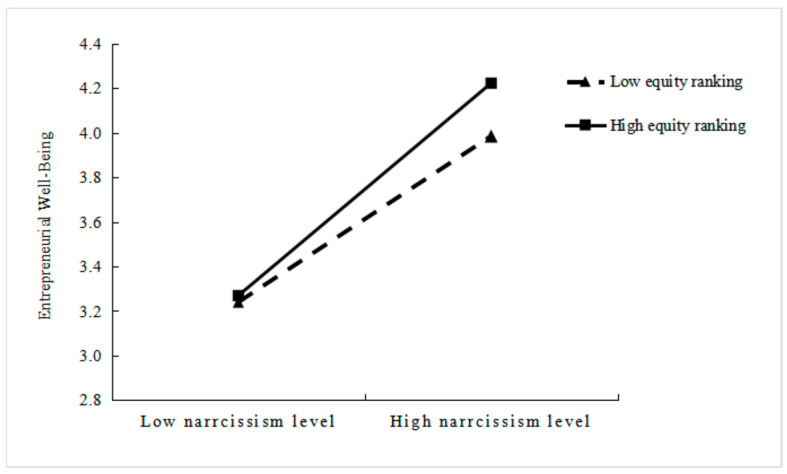
The Moderating Role of Equity Ranking in the Narcissism–Well-Being Link.

Figure 1 graphically illustrates the moderating effect of equity ranking on the relationship between narcissism and well-being. The Figure 1 shows that the positive influence of equity ranking on well-being is amplified under conditions of high equity ranking (i.e., holding the top position in equity ranking). The positive effect of equity ranking on well-being is attenuated under conditions of low equity ranking (i.e., holding a non-first position in equity ranking). Consequently, this representation provides empirical support for Hypothesis 2.

**Figure 2:**
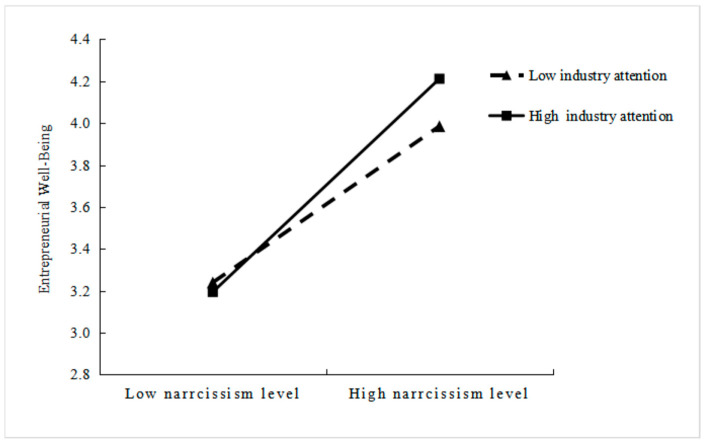
The Moderating Role of Industry Attention in the Narcissism–Well-Being Link.

Figure 2 delineates the moderating effect of industry attention on the relationship between narcissism and well-being. The figure illustrates that the positive influence of industry attention on well-being is intensified under conditions of high industry attention. The positive effect of industry attention on well-being is diminished under conditions of low industry attention. This representation offered evidence supporting Hypothesis 3.

### 4.3. Robustness Tests

To ensure the robustness of the findings, this study employed two complementary methods for robustness testing: variable substitution and sub-sampling. First, narcissism was converted into a binary variable based on its mean value (0.406), allowing us to categorize it into high (coded as 1) and low (coded as 0) levels. Subsequently, regression analyses were conducted using this dummy variable in place of the original independent variable. The regression coefficients maintained their significance levels, indicating the stability of the results. Details are given in Table 5.

Second, drawing on previous research, we excluded entrepreneurs with less than one year of work experience from our sample. This is based on the recognition that entrepreneurs in the early stages of their careers often lack a strong sense of self. As a result, this exclusion did not diminish the significance of the main effects; the results remained congruent with those from the unfiltered sample. Details are provided in Table 6.

## 5. Discussion

### 5.1. Theoretical Contributions

Well-being serves as a multifaceted indicator. It reflects an individual’s intrinsic psychological needs and serves as a measure for entrepreneurs pursuing self-actualization. Previous research has predominantly focused on the relationship between entrepreneurial activities and need satisfaction from a motivational perspective; however, fewer studies have explored the role of personality traits in this relationship. To fill this gap, this study adopts entrepreneurs’ personality traits as a foundational framework. This study broadens the research landscape regarding well-being in entrepreneurship.

First, this study examines well-being from a personality-centric perspective, thereby extending the understanding of well-being in entrepreneurial contexts. According to Deci and Ryan (2000), well-being arises from satisfying an individual’s core needs. Prior studies typically followed the trajectory of “entrepreneurship-need fulfillment-well-being” (Deci & Ryan, 2000; Shepherd & Cardon, 2009). However, differences in personality traits not only engender cognitive disparities but also signify variations in entrepreneurial motivations and needs (Liu et al., 2019). This study aims to extend previous research by focusing on the relationship between narcissistic personality and well-being.

Second, by investigating the subjective experiences of narcissistic entrepreneurs during their entrepreneurial journeys, this study provides deeper insights into the role of narcissism in entrepreneurship. Narcissism is identified as a significant entrepreneurial trait and a motivating force (Navis & Ozbek, 2016). Consequently, a corpus of research has emerged regarding the association between narcissistic personality and entrepreneurship (Mathieu & St-Jean, 2013; Yu et al., 2020; Wales et al., 2013a). These studies reveal that individuals with high levels of narcissism are more likely to engage in entrepreneurial activities. These studies offer insights into their distinctive behavioral patterns. By exploring their emotional experiences, we can better understand the motivations that drive their entrepreneurial persistence. Therefore, this study presents a more comprehensive perspective on narcissistic entrepreneurship and advances research on narcissistic personality in entrepreneurial contexts.

Furthermore, the study uncovers variations in the relationship between narcissism and well-being, contingent upon different contexts. This finding highlights the importance of considering contextual factors when examining the interplay between personality traits and individual well-being. This study examines the moderating effects of factors on the relationship between narcissism and well-being: entrepreneurial firm characteristics and industry characteristics. It further corroborates that well-being is a result of the alignment between individual attributes and situational factors. In other words, when entrepreneurs’ core needs are met in their environment, they are likely to experience higher levels of well-being.

### 5.2. Practical Contributions

The findings reveal that entrepreneurs with pronounced narcissistic traits often experience higher levels of well-being during the entrepreneurial process. This finding suggests that personality traits influence personal development. Specifically, the study highlights the role of narcissism in shaping entrepreneurs’ well-being, providing a new perspective on self-awareness and psychological resilience. Entrepreneurs can utilize tools such as self-assessments and interpersonal communication to identify their personality traits and align their entrepreneurial endeavors with their strengths, fostering positive experiences.

Furthermore, from an organizational management perspective, insights from this study can refine leaders’ interactions with their teams. Involving narcissistic employees in decision-making and providing them with opportunities to express their views can increase their motivation and satisfaction. This approach can enhance team cohesion and efficiency, ultimately fostering organizational growth.

Finally, this study aims to raise awareness and support for entrepreneurs in the wider community. This can be achieved by raising the awareness of the public about entrepreneurs through the media and public events. This aims to satisfy the need for external attention by making the society at large aware of the value and the contribution of entrepreneurs.

### 5.3. Limitations and Future Directions

Based on a sample of Chinese technology entrepreneurs, this study found a significant positive correlation between narcissism and well-being. This finding aligns with the results of Leung et al. (2021) based on multi-country data, suggesting that the entrepreneurial context may amplify the adaptive function of narcissism. Specifically, the autonomy, decision-making control (reflected by high equity ranking), and potential public attention (reflected by high industry attention) provided by entrepreneurial activities correspond closely to the core needs of highly narcissistic individuals for power and self-display. This alignment may facilitate the transformation of their personality traits into greater work enthusiasm and a sense of accomplishment (Mathieu & St-Jean, 2013).

However, the relationship between narcissism and well-being must be viewed dialectically. Although this study and Leung et al. (2021) identified a positive association in the entrepreneurial context, extensive literature also highlights potential threats that narcissism may pose to well-being. Narcissistic traits, particularly the ‘entitlement/exploitativeness’ dimension, are often accompanied by a lack of empathy and an instrumental approach to relationships (Ackerman et al., 2011). This behavioral pattern can impair the quality of their relationships with partners, team members, and even family (Brunell & Campbell, 2011). Future research should longitudinally track entrepreneurs to examine whether the negative effects of narcissism become more pronounced under adverse conditions, such as team conflict or venture failure.

Despite offering a novel perspective on understanding entrepreneurial well-being, this study has several limitations. First, although archival data were used to reduce common method bias, equity rankings are static cross-sectional indicators that cannot reflect dynamic changes. While the Baidu Index effectively captures online attention, its ability to represent deeper, offline forms of industry attention may be limited. Future research could incorporate real-time tracking data to more dynamically capture the influence of contextual factors.

Second, this study is primarily based on cross-sectional data, providing evidence of the association between narcissism and well-being among operating business owners at a single point in time. It does not fully capture the dynamic process within the entrepreneurial ladder (Van der Zwan et al., 2010). The cross-sectional design cannot establish causality and does not account for how the entrepreneurial context evolves over time. Adopting a longitudinal research design would help elucidate the trajectory of well-being over time and the mechanisms through which situational factors operate.

Furthermore, due to the limited sample source (Chinese technology entrepreneurs), the generalizability of the findings should be interpreted with caution. There may be other individual or environmental variables that affect well-being that are not included in the model. Factors such as cultural background and socioeconomic context may influence entrepreneurs’ experiences of well-being. Future research should expand the sample scope to include entrepreneurs from diverse regions, industries, and cultural backgrounds to enhance the reliability and external validity of the results.

## 6. Conclusions

This study addresses two key questions: “Why do some entrepreneurs experience higher levels of well-being?” and “When do entrepreneurs feel happier?” It examines the relationship between narcissism and well-being, as well as the moderating effects of situational characteristics. Through a multi-method approach combining survey responses from 165 entrepreneurs with archival data on equity rankings and industry attention, we reached several key conclusions.

First, a positive association was found between narcissistic traits and well-being. This finding is consistent with the view that entrepreneurial activities may provide experiences of attention and control that are particularly fulfilling for individuals with narcissistic tendencies.

Second, the positive association between narcissism and well-being was stronger among entrepreneurs with a top equity ranking. This pattern aligns with the notion that greater decision-making authority (associated with high equity) may better satisfy the need for control, thereby intensifying the observed association.

Finally, the association was also stronger in industries receiving high levels of public attention. This result supports the idea that environments rich in external recognition opportunities may strengthen the positive link between narcissism and well-being.

## Figures and Tables

**Table 1:** Descriptive Statistics of Entrepreneur and Firm Characteristics.

Variable	Item	Sample Size	Ratio Percent	Mean	SD
Municipalities	Beijing	46	27.9	-	-
	Fujian	12	7.3	-	-
	Guangdong	39	23.6	-	-
	Shandong	59	35.8	-	-
	The rest	9	5.5	-	-
Sex	Female	36	21.8	-	-
	Male	129	78.2	-	-
Age	≤25	9	5.5	-	-
	26~40	80	48.5	-	-
	41~55	69	41.8	-	-
	≥55	7	4.2	-	-
Education	High school	4	2.4	-	-
	Junior college	20	12.1	-	-
	Undergraduate education	88	53.3	-	-
	Master	39	23.6	-	-
	Doctor	14	8.5	-	-
Entrepreneurial experience	1 time	95	57.6	-	-
	2 times	36	21.8	-	-
	3 times	27	16.4	-	-
	>3 times	7	4.2	-	-
Entrepreneurial Age	-	-	-	4.8	4.6
Number of team members	-	-	-	7.2	7.8

**Table 2:** Industry Attention.

Industry Name	Industry Attention
New energy industry	970
New generation of information technology industry	783
New materials industry	598
Biology industry	346
High-end equipment manufacturing industry	283
Software and information technology services industry	209
Business services industry	162
Science and technology promotion and application services industry	84

**Table 3:** Descriptive Statistics and Correlations for All Variables.

	1	2	3	4	5	6	7	8	9	10	11
1 Well-being	1										
2 Narcissism	0.349 **	1									
3 Equity ranking	0.205 *	0.166	1								
4 Industry attention	0.131	0.055	−0.022	1							
5 Education	−0.018	0.213 **	0.002	0.098	1						
6 Self-efficacy	0.419 **	0.164 *	0.152	0.102	0.115	1					
7 Empathy	0.315 **	−0.031	0.084	0.004	0.142	0.376 **	1				
8 Work experience	0.119	−0.044	0.121	0.069	0.022	0.085	−0.057	1			
9 Performance	−0.038	0.057	−0.065	0.300 **	0.159	0.155	0.087	0.070	1		
10 Entrepreneurial experience	0.151	0.141	−0.042	0.059	−0.060	−0.050	−0.017	−0.025	−0.008	1	
11 Types of entrepreneurship	0.107	0.268 **	0.022	−0.056	0.029	0.176 *	0.049	−0.094	0.068	0.116	1
Mean	5.507	0.406	0.838	0.147	3.213	3.103	3.165	10.096	32.607	1.699	0.685
SD	0.904	0.202	0.370	0.355	0.864	0.507	0.685	6.636	76.507	0.988	0.466

Note: * and ** indicate *p* < 0.05 and *p* < 0.01, respectively.

**Table 4:** Hierarchical Regression Analysis Results for Narcissism, Moderators, and Entrepreneurial Well-being.

	Model 1	Model 2	Model 3
Education	−0.068 +	−0.147 +	−0.169 *
Self-efficacy	0.332 **	0.233 **	0.276 **
Empathy	0.210 **	0.365 **	0.371 **
Work experience	0.114	0.142 *	0.161 *
Entrepreneurial experience	0.164 *	0.131 *	0.159 *
Performance	−0.082	−0.095	−0.134 *
Types of entrepreneurships	0.035	−0.043	−0.037
Narcissism		0.370 **	0.120 **
Equity ranking		0.088	0.135 +
Industry attention		0.093	0.095
Narcissism × Equity ranking			0.123 +
Narcissism × Industry attention			0.128 +
F	7.243 **	9.724 **	8.876 **
R2	0.244	0.387	0.412
Adj.2	0.210	0.347	0.366

Note: +, *, and ** denote *p* < 0.1, *p* < 0.05, and *p* < 0.01, respectively. We conducted tests for multicollinearity by calculating the Variance Inflation Factor (VIF). The maximum VIF in all models was [1.344], well below the threshold of 5, indicating that multicollinearity is not a concern.

**Table 5:** Robustness tests for replacing key variables.

	Model 1	Model 2	Model 3
Control variable	Containment	Containment	Containment
Narcissism		0.342 **	0.333 **
Equity ranking		0.130 +	0.168 *
Industry attention		0.068	0.046
Narcissism × Equity ranking			0.141 +
Narcissism × Industry attention			0.114 +
F	7.243 **	9.213 **	8.462 **
R2	0.244	0.374	0.400
Adj.2	0.210	0.334	0.353

Note: +, *, and ** denote *p* < 0.1, *p* < 0.05, and *p* < 0.01, respectively.

**Table 6:** Robustness test for sub-samples.

	Model 1	Model 2	Model 3
Control variable	Containment	Containment	Containment
Narcissism		0.364 **	0.404 **
Equity ranking		0.088	0.135 +
Industry attention		0.097	0.098
Narcissism × Equity ranking			0.126 +
Narcissism × Industry attention			0.131 +
F	6.773 **	9.016 **	8.275 **
R2	0.238	0.377	0.403
Adj.2	0.203	0.335	0.354

Note: +, and ** denote *p* < 0.1, and *p* < 0.01, respectively.

## Data Availability

The data of this study is available from the corresponding author upon reasonable request.
